# Histological Evaluation of Gonad Impairments in Russian Sturgeon (*Acipenser gueldenstaedtii*) Reared in Recirculating Aquatic System (RAS)

**DOI:** 10.3390/ani10081439

**Published:** 2020-08-18

**Authors:** Małgorzata Rzepkowska, Dobrochna Adamek-Urbańska, Magdalena Fajkowska, Marek Łukasz Roszko

**Affiliations:** 1 Department of Ichthyology and Biotechnology in Aquaculture, Institute of Animal Sciences, Warsaw University of Life Sciences, 02-786 Warsaw, Poland; dobrochna_adamek@sggw.edu.pl (D.A.-U.); magdalena_fajkowska@sggw.edu.pl (M.F.); 2 Department of Food Analysis, Institute of Agricultural and Food Biotechnology, 02-532 Warsaw, Poland; marek.roszko@ibprs.pl

**Keywords:** follicular atresia, gonadal disorders, intersex gonad, ovarian fat, recirculating aquatic systems, sturgeon

## Abstract

**Simple Summary:**

The growing interest in sturgeon aquaculture necessitates the constant development of rearing techniques with special emphasis on the maintenance of high fish reproduction performance. However, there is a substantial lack of knowledge regarding gonadal impairments in sturgeons, even though the occurrence of such disorders poses a significant threat to the future of both commercial and conservation farming. Therefore, the aim of the research was to determine potential pathologies in the gonads of over-four-year old Russian sturgeon (*Acipenser gueldenstaedtii*) reared in the controlled conditions of recirculating aquatic systems (RAS). RAS have gained popularity in recent years, as they are considered the most environmentally friendly way of producing fish. Nevertheless, there are many issues that need to be resolved regarding sturgeon RAS farming, including its effect on gonadal development and, therefore, fish reproduction capacity. A detailed histological analysis revealed multiple gonadal disorders in the analyzed sturgeon, some of which can contribute to decreased fish fertility or even sterility. The female-biased sex ratio and the character of the identified impairments indicates that the observed germinal tissue disruptions might originate from estrogenic endocrine disturbances. Therefore, for the further sustainable development of sturgeon RAS aquaculture, there is an urgent need to identify and eliminate the causes of such disruptions.

**Abstract:**

The aim of the study was to raise an issue concerning gonadal impairments in sturgeon reared in recirculating aquatic systems (RAS). In the present study, an in-depth histological evaluation in terms of gonadal pathologies was performed on over-4-year-old (1600 days post-hatching) Russian sturgeon (*Acipenser gueldenstaedtii*) reared under indoor RAS. A female-biased sex ratio, intersex occurrence, ovarian fat overgrowth, T-cell infiltration and follicle atresia were the most commonly observed disorders in the analyzed gonads. The combined processes of oocyte autophagy and follicular cell apoptosis were engaged in follicular atresia; however, atretic follicles showed a varied morphology, whereas oogonia and oocytes in the early stages of meiosis, as well as spermatogonia, underwent degeneration by apoptosis. The most severe pathology was observed in females with abundant intra-ovarian fat deposition. The extremely fatty ovaries were noted to lose the majority of ovarian follicles, which directly leads to fish sterility. The identified impairments might be related to estrogenic endocrine disruption, as feminization and unspecific vitellogenin synthesis were detected, although the sources of the observed pathologies can be diverse. Therefore, the presented research lays the groundwork for further studies on reproductive disorders in this prized and endangered fish species.

## 1. Introduction

From an economic point of view, sturgeons are fish of great value, being a source of caviar and meat production. Over the years, wild sturgeon populations have diminished greatly due to overfishing and poaching combined with water pollution and a loss of natural habitats. This has resulted in the current status of the *Acipenseridae* family as one of the most endangered among all animal species [1]. The Russian sturgeon (*Acipenser gueldenstaedtii*), which originated in the Caspian Sea basin, is one of these critically endangered species and has relatively recently been introduced into Western European aquaculture [2].

Recirculating aquatic systems (RAS) are considered as the most environmentally friendly way of producing fish at a commercially viable level, and sturgeons are one of the species of interest to be reared under RAS conditions [3]. RAS have already been successfully applied in hatcheries designed to produce sturgeons for restocking [4]; however, they can also be attractive for intense sturgeon production to market size, especially in countries with strong environmental regulations, either on outlet water quality or on non-native species farming [5]. To date, however, there has been insufficient knowledge on the optimal conditions in terms of sturgeon RAS rearing, and general recommendations for sturgeon farming are often incomplete or inconsistent. Sturgeons easily adapt to the RAS environment [6], showing high adjustability regarding a wide range of temperature levels, i.e., 11–26 °C [7,8], and generally tolerating unusual rearing conditions [9]. The highest sensitivity of sturgeons reared in RAS was noted in terms of oxygen and ammonia levels [10,11]. The general recommendations concerning these parameters are oxygen (D.O.) < 6 mg L^−1^; pH: 7.0–8.0; total ammonia nitrogen (TAN): adult < 2.0 mg/dm^3^, juveniles < 0.005 mg/dm^3^; NO_2_–N: <0.50 mg/dm^3^; NO_3_–N: <25 mg/dm^3^ [8,10,12]. The optimal sturgeon stocking density was estimated at 80–90 kg/m^3^ [8]. Light intensity and photoperiod, except constant light, have no effect on fish growth [13,14]. Formulated diets can be used to feed most sturgeon species [15], although information on sturgeon nutrient requirements is urgently needed for success in culturing these species of fish [16].

Over the last 20 years, the number of data on sturgeon gonadal development has increased rapidly [17]; however, the knowledge about the pathologies in this process is very limited, which makes disturbed gametogenesis difficult to recognize. Moreover, there is a substantial lack of information concerning the impact of particular rearing parameters on sturgeon gonad differentiation and reproduction capacity. Bahmani et al. [18] suggested that thermo-hydro-biological conditions play a crucial role in sturgeon gonadal development, and Webb et al. [19] demonstrated that exposure to a constant temperature prior to sturgeon spawning can negatively affect the late vitellogenic and post-vitellogenic phases of ovarian development, leading to follicular atresia. However, rearing the sturgeon in year-round warm water accelerates their growth and sexual maturity and does not have an adverse effect on ovarian vitellogenesis [20]. Elevated nitrate levels were shown to be capable of altering the steroid profiles of cultured female Siberian sturgeon (*Acipenser bareii*), as well as causing secondary stress responses, yet more research needs to be done to evaluate the impact of this pollutant on reproductive performance in sturgeons [21]. High stocking density was shown to cause stress in stellate sturgeon (*Acipenser stellatus*), which was manifested by a decrease in testosterone plasma concentrations. In this study, poor-quality sturgeon male gametes were also positively correlated with chronic stress, caused by prolonged hatchery practices in high water temperatures [22]. The annual photoperiod is likely to control gametogenesis in sturgeons, although no experimental evidence has been available to date [23]. One of the environmental factors that has lately been shown to have a profound effect on fish reproduction is hypoxia, causing, among others, testicular and ovarian development inhibition [24]. To our knowledge, there are no data concerning the effect of oxygen level on gonad development in sturgeons; however, it is known that sturgeons are unusually sensitive to hypoxic conditions [25].

In the present research, the Russian sturgeon were reared for over 4 years (1600 days) in indoor RAS. Throughout this period, fish from this stock were extensively studied with respect to gonadal development and differentiation. The results obtained from the period covering the first 800 days of rearing demonstrated ongoing feminization and the development of intersex gonads in the analyzed stock [26]. Therefore, the aim of this study was to describe all observed pathologies in the gonads of the Russian sturgeon after 1600 days of RAS rearing, laying the groundwork for further studies on reproductive disorders in this prized and endangered fish species.

## 2. Materials and Methods

### 2.1. Rearing Conditions

Larvae of the Russian sturgeon, progeny of several individuals from different crosses, were obtained from the fish farm on the 3rd day post-hatching (dph) and were reared up to 4 years (1600th dph) in indoor RAS tanks, supplied with filtered tap water. The larvae were kept initially in 5 dm^3^ aquaria placed into RAS tanks of 225 dm^3^ capacity. On the 30th dph, fish were transferred directly into RAS tanks. After the 200th dph, the fish were transferred into tanks of 1150 dm^3^ capacity, and after the 800th dph, into tanks of 1440 dm^3^ capacity, where they were reared to reach 4 years of age. The stocking density did not exceed recommended values for sturgeon species during the entire rearing period [27]. Each RAS was equipped with automatic water oxygen aeration (OXYMAT, Helsinge, Denmark), mechanical and biological filters and a UV-disinfection system. A constant 12 h light/12 h dark photoperiod was applied over the entire rearing period. The temperature (20.3 ± 0.7 °C), pH (7.0–7.5) and dissolved oxygen concentration (D.O.) (7.6 ± 0.2 mg/dm^3^) in the aquaria and indoor recirculating tanks were kept at a constant level, according to Rzepkowska and Ostaszewska [28]. The TAN (total ammonia nitrogen) and NO_3_-N did not exceed 0.6 and 8 mg/dm^3^, respectively.

### 2.2. Feeding Regime

After yolk-sac absorption and melanin plug ejection at the 8th dph, the Russian sturgeon larvae were fed ad libitum manually with live brine shrimp Artemia sp., mixed with the Perla Larva commercial feed, at the approximated level of 15–30% of fish biomass per day (Skretting, Stavanger, Norway). At the 20th dph (average fry weight, 0.16 g), the feed was gradually changed to a commercial diet only. From the 30th dph when the fry reached an average weight of 0.5 g, Nutra (Skretting, Stavanger, Norway) feeds were administered, and when the fingerlings reached 70 g (around the 200th dph), Estella (Skretting, Stavanger, Norway) feeds were applied, in granulation adequate for fish size. The feeds were administered by automatic feeders: from the 30th to the 35th dph in ten, from the 35th to the 60th dph in seven, from the 60th and the 200th dph in five and from the 200th to 1600th dph in four equal doses per day. More detailed information on the feeds is provided in Rzepkowska et al. [29]. The survival rate over the first 30 dph was 70.7%; at the 800th dph, mortality was below 5%. Later on, no mortality occurred.

### 2.3. Sample Collection and Procedures

Out of 60 Russian sturgeon reared in three separate RAS tanks at 1600th dph, five individuals (TL = 83.4 ± 6.8 cm; W = 5287.5 ± 328.2 g) from each tank were selected for the histological and immunohistochemical (IHC) evaluation of the gonadal tissue. Before sampling, the fish were starved for 24 h, then anaesthetized with MS-222 (tricaine methanesulphonate, Sigma-Aldrich, St. Louis, MO, USA) and euthanized by decapitation. The procedure was conducted in accordance with the protocol accepted by the II Local Ethic Committee for Animal Research (Warsaw, Poland) of the permission no. WAW2/42/2017 of 8 May 2017. Dissected gonads were cut into small pieces, which were randomly chosen for analyses. A standard histological procedure was applied to prepare Bouin-fixed paraffin embedded (BFPE) and formalin-fixed paraffin embedded (FFPE) samples, which were cut into 5 µm-thick sections using a Leica RM2265 microtome (Leica Microsystems, Nussloch, Germany).

### 2.4. Histological and Immunohistochemical (IHC) Analysis

The sex of the specimens and the topology of the gonadal tissue were assessed based on the analysis of the BFPE sections stained with hematoxylin–eosin (H-E), Masson trichrome, Azan trichrome, Alcian Blue/PAS (AB/PAS) and PAS with Weigert’s iron hematoxylin methods. Stages of gonadal development were assigned in accordance with Webb et al. [17]. Apoptosis was detected with the TUNEL method using an ApopTag Peroxidase In Situ Apoptosis Detection Kit S7100 (Millipore, Burlington, MA, USA) on the FFPE sections using terminal deoxynucleotidyl transferase (TdT) in a 1:3 dilution. The method was applied with modifications: proteinase K digestion was omitted, and counterstaining in 0.5% water Eosin Y solution was applied. The positive control was digested with 50 µL of DNase I recombinant (2500 U/mL) (Roche, Basel, Switzerland), and in the negative control, TdT was omitted. For vitellogenin detection, monoclonal, mouse, anti-gulf sturgeon vitellogenin antibody (ND-1H2, Cayman Chemical Company, Ann Arbor, MI, USA) in a 1:500 dilution was applied. Incubation with anti-vitellogenin antibody was performed overnight at 4 °C. T-cells (Lymphocytes T CD3+) were detected using a mouse anti-human CD3 monoclonal ready-to-use antibody solution (Bond™ Ready-to-Use Primary Antibody CD3, LN10, Leica, Newcastle, UK). Incubation with the anti-CD3 antibody was performed for 30 min at RT. Reactions with both antibody types were performed on BFPE sections. A citrate buffer was used for Heat-Induced Epitope Retrieval (HIER). A Peroxidase Detection System (Leica, Newcastle, UK) was applied for visualization in accordance with manufacturer recommendations. The specificity of the applied antibodies was tested by Western-Blot analysis. In the negative control, incubation with the primary antibody was omitted.

### 2.5. Microscopic, Histometric and Statistical Analyses

Microscopic and histomorphometric analyses were performed using a Nikon ECLIPSE 90i microscope (Tokyo, Japan) and the NIS-Elements AR 5.01.00 computer image analysis system (Nikon Corporation, Tokyo, Japan). The number of ovarian follicles per 1 mm^2^ and the percentage of early atretic follicles were analyzed on the folded surface of the ovaries, which contained pre-vitellogenic oocytes (second stage of ovarian development in accordance with Webb et al. [17]). Normal and early atretic perinucleolar oocytes were counted in ten random areas of at least three longitudinal sections of each gonad. The advanced and final stages of follicular atresia were not analyzed due to highly uneven distributions throughout the analyzed areas. The percentage of spermatogenic tubules was evaluated on the frontal plane of the ventral gonadal surface. If the female component occupied the ventral surface of the gonad, random areas across the gonad were analyzed, excluding the dorsal (distal) tubules. Ten random areas in each of at least three sections per one specimen were evaluated. The spermatogonia percentage was referred to the 1 mm^2^ of the seminiferous tubule area. The percentage of spermatogonia and spermatogonia degeneration was analyzed in 20 pre-meiotic seminiferous tubules for each of the analyzed areas of the testicular component of individuals at the pre-meiotic and onset-of-meiosis stages (second and third stages of testicular development in accordance with Webb et al. [17]). The data are presented as mean ± one standard deviation (SD). Statistical differences between individual measurements were calculated by one-way ANOVA with the NIR Fisher post-hoc test using the Statistica 13 software (StatSoft, Tulsa, OK, USA).

## 3. Results

### 3.1. Sex Ratio and Gonad Anatomo-Morphology

**Figure 1:**
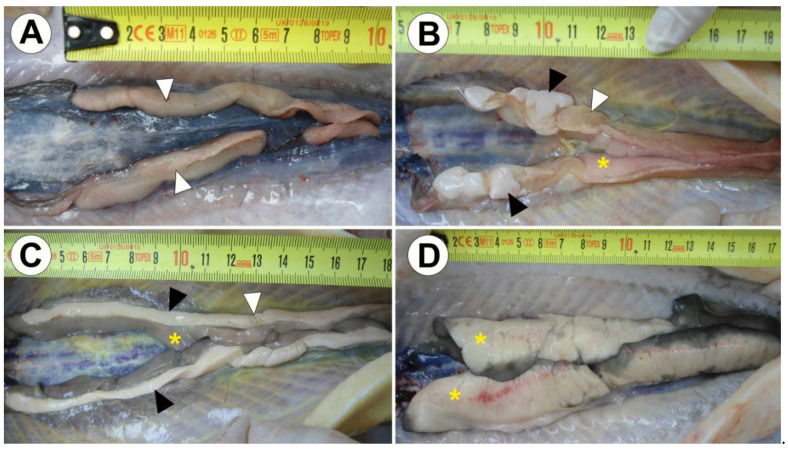
Gonads of 1600th-day-post-hatching (dph) Russian sturgeon. (**A**) Ovary with folded and granular female germinal tissue. (**B**) Ova-testes with a prevailing ovarian component located in the posterior part of the gonad, and testicular component located in the anterior part of the gonad. (**C**) Testes-ova with a prevailing testicular component and an ovarian component scattered apically along the gonad surface. (**D**) Ovary with abundant, white and locally pigmented fat tissue (presence of the germinal tissue on the surface of gonadal folds was only possible to assess by microscopic analyses). Ovarian germinal tissue (white arrowheads), testicular germinal tissue (black arrowheads) and fat (yellow stars).

Among the 15 analyzed Russian sturgeon, nine specimens were identified as females; five, as intersex; and only one, as male. In all of the analyzed Russian sturgeon specimens, the gonads were well differentiated. They extended along the dorsal body cavity, however, not always continuously (Figure 1A). In the anterior part, the gonads were spaced ventrally, while in the posterior part, they ran parallel dorsally. The ovaries or ovarian areas in the intersex specimens were yellowish, lamellar and granular on the surface (Figure 1A,B), while the testes or testicular regions were pale white and smooth (Figure 1B,C). Among the analyzed intersexes, different types of gonads were recognized: ova-testes with well-developed male and female areas (Figure 1B) and testes-ova with narrow female regions distributed alongside on the testicular surface (Figure 1C). The amount of gonadal fat was vastly variable, ranging from nothing (Figure 1A), to occupying all of the gonadal lobes (Figure 1D).

### 3.2. Ovarian Histomorphology and Histopathology

**Figure 2:**
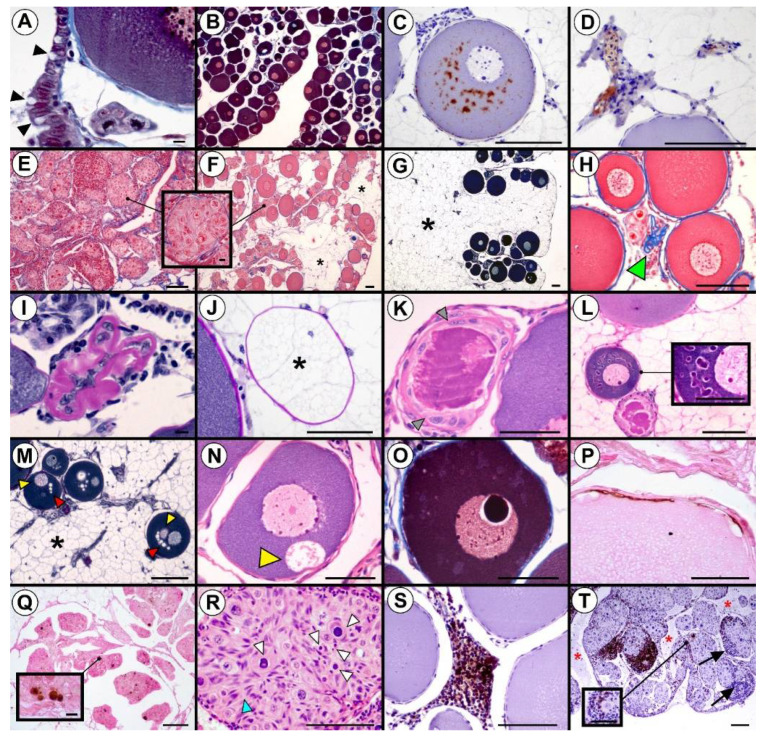
Ovaries of 1600th-day-post-hatching (dph) Russian sturgeon. (**A**) Columnar epithelium in the furrows of the folds. (**B**) Growing ovarian follicles containing perinucleolar oocytes in primary growth stage. (**C**) Deposition of vitellogenin (brown) in ooplasm of small perinucleolar oocyte and (**D**) blood vessel plasma. (**E**) Nests of oogonia and oocytes at the chromatin-nucleolus stage. (**F**) Ovarian nests among sparse ovarian follicles. Magnification between (**E**) and (**F**): Oogonia and oocytes at the chromatin-nucleolus stage inside the ovarian nest. (**G**) White fat tissue in the germinal region of the gonad. (**H**) Final phase of follicular atresia. (**I**) Residual follicular cells and (**J**) adipocytes inside a completely phagocytized follicle. (**K**) Advanced stage of follicular atresia. (**L**) Normal ovarian follicle (at the top), early stage of follicular atresia (in the middle) and advanced stage of follicular atresia (at the bottom). Magnification inside (**L**): Heterogeneous ooplasm of degenerating perinucleolar oocyte. (**M**) Vacuoles and (**N**) autophagosomic bodies within the ooplasm of degenerating perinucleolar oocytes at an early stage of follicular atresia. (**O**) Large vesicles in the nuclei of degenerating perinucleolar oocytes at early stage of follicular atresia. (**P**) Follicular cell apoptosis (brown nuclei). (**Q**) and magnification inside: Apoptosis of oogonia and chromatin-nucleolus oocytes (brown nuclei). (**R**) Nest with multiple atretic oogonia and oocytes at the chromatin-nucleolus stage. (**S**) Small cluster of lymphocytes (**T**) (dark brown) between ovarian follicles. (**T**) and magnification inside: Oogonia and primary chromatin-nucleolus stage oocyte nests infiltrated by T- (dark brown) and B-cells (blue). **Indicators:** secretory cells resembling goblet cells (black arrowhead), adipocytes (black stars), basement membrane (green arrowhead), hypertrophied follicular cells (grey arrowheads), autophagosomic bodies (yellow arrowheads), vacuoles (red arrowheads), prefollicular cells (blue arrowhead), pyknotic nuclei at different stages of oogonia/chromatin-nucleolus oocyte atresia (white arrowhead), B lymphocytes (black arrows), and interstitial and superficial fluid containing leukocytes (red stars). **Staining:** Masson trichrome (**A**,**B**,**O**), immunohistochemical (IHC)—anti-vitellogenin antibody (**C**,**D**), Azan trichrome ((**E**,**F**) and magnification in between, (**H**)), PAS with Weigert’s iron hematoxylin (**G**,**M**), AB/PAS (**I**,**J**), H-E ((**K**,**L**) and magnification inside, (**N**,**R**)), TUNEL ((**P**,**Q**) and magnification inside), IHC—anti-CD 3 antibody ((**S**,**T**) and magnification inside (**T**)). **Scale bars**: (**A**), magnification between (**F**) and (**E**,**I**,**M**) and magnification inside (**Q**) = 10 µm; (**B**–**H**,**L**,**Q**–**T**) = 100 µm; (**J**,**K**,**N**–**P**) and magnification inside (**L**,**T**) = 50 µm.

The ovaries of the Russian sturgeon on the 1600th dph were lamellar, covered with flat epithelium on the top of the folds and columnar epithelium in the furrows. In the covering epithelium in one of the analyzed females, secretory cells resembling goblet cells were observed (Figure 2A). The female germinal tissue in most of the analyzed individuals was mainly composed of ovarian follicles in which pre-vitellogenic, perinucleolar oocytes in the primary growth phase were arrested in the dictyotene of the meiotic prophase I (Figure 2B) (Table 1). However, in two out of the nine analyzed females, a vitellogenin-positive reaction was observed in the ooplasm of a few small, perinucleolar oocytes (Figure 2C) (Table 1). Additionally, vitellogenin was also detected in the plasma inside the ovarian blood vessels of those specimens (Figure 2D) (Table 1). In two other females, delayed oogenesis was observed, characterized by the dominance of nests with oogonia and early meiotic oocytes at the chromatin-nucleolus stage. In the gonads of the first one, only nests of oogonia and chromatin-nucleolus oocytes were present, while in the second, growing follicles containing perinucleolar oocytes were also observed, although diffusely distributed (Figure 2E,F) (Table 1).

Interstitial (intraovarian) white fat tissue in the germinal region of the gonad was recognized in eight out of the nine females (Table 1). Adipocytes occupied the dorsal and the central part of the ovarian folds, while ovarian follicles or ovarian nests were located apically, mostly in the furrows of folds (Figure 2F,G). In the ovaries with abundant white fat tissue, a profound reduction in the number of ovarian follicles was observed, although follicular atresia was noted in all of the analyzed females (Table 1). Completely phagocytized follicles in the final stage of atresia were characterized by the presence of collapsed basement membranes (Figure 2H,I), inside of which residual follicular cells (Figure 2I) or adipocytes were often observed (Figure 2J). In the advanced stage of follicular atresia, pronounced hypertrophy of follicular cells was associated with the heterophagocytosis of the oocyte content (Figure 2K). Inside such atretic follicles, residual eosinophilic ooplasm of degenerating oocytes was still evident (Figure 2K,L). In the early stages of follicular atresia, perinucleolar oocytes showed diversified morphology. The most common feature of this atresia stage was distinctly darker and heterogenous oocyte ooplasm (Figure 2L), in which vacuoles (Figure 2M) and autophagosomic bodies (Figure 2M,N) were often present. In the germinal vesicles (nuclei) of early atretic perinucleolar oocytes, one or a few large vesicles, resembling large nucleoli, were also commonly observed (Figure 2O). However, these features did not occur simultaneously in all degenerating oocytes. In larger follicles, containing oocytes in the perinucleolar stage, atresia occurred due to the apoptotic process of the follicular cells (Figure 2P) and not due to the apoptosis of oocytes, as their nuclei were always TUNEL-negative. In contrast, oocytes at the chromatin-nucleolus stage and oogonia underwent atresia by apoptosis, which was particularly severe in the female containing only ovarian nests (Figure 2Q). Such nests were characterized by the presence of multiple hypertrophic prefollicular cells and numerous dark pyknotic nuclei (Figure 2R).

Lymphocyte infiltration into ovarian germinal tissue was observed in five females (Table 1). In four females, lymphocytes, which were generally T-cells (CD3+), were clustered in a small area (Figure 2S). In the female containing exclusively ovarian nests, both T- and B-cells were present, penetrating a large germinal tissue area. In the ovarian tissue of this female, large amounts of interstitial and superficial fluid containing other leukocytes were also observed (Figure 2T).

### 3.3. Testicular and Intersex Gonad Histomorphology and Histopathology

**Figure 3:**
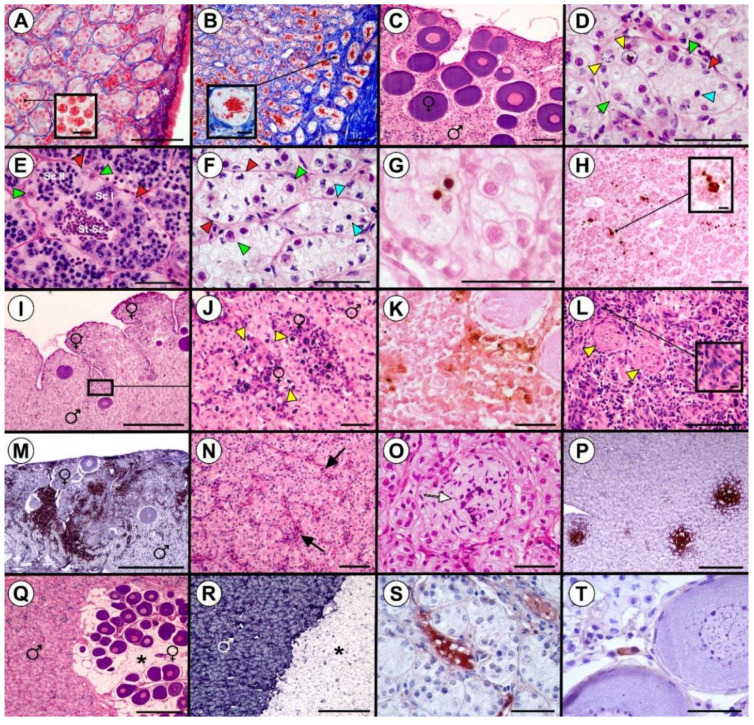
Testis (**A**,**B**,**P**) and intersex gonads (**C**–**O**,**Q**–**T**) of 1600th-day-post-hatching (dph)Russian sturgeon. (**A**) Low columnar epithelium on the testicular surface with a thin tunica albuginea and seminiferous tubules underneath. Magnification inside (**A**): Primary spermatocytes. (**B**) and magnification inside: Accumulation of spermatozoa (red) inside distal seminiferous tubules. (**C**) Intersex gonad with ovarian follicles located ventrally (female symbol ♀) and seminiferous tubules located dorsally (male symbol ♂). (**D**) Chromatin-nucleus oocytes inside seminiferous tubule. (**E**) Spermatogenesis in cysts of an enlarged seminiferous tubule. (**F**) Pre-meiotic seminiferous tubules with low spermatogonia density. (**G**) Apoptotic degeneration of spermatogonia in a pre-meiotic seminiferous tubule. (**H**) Severe apoptosis in meiotic seminiferous tubules. Magnification inside (**H**): Apoptotic clusters of spermatogenic cells inside seminiferous tubules. (**I**,**J**) Chromatin-nucleus oocytes located in the folded ventral area of the gonad. (**K**) Increased apoptosis in the feminizing area of the intersex gonad. (**L**) and magnification within: Infiltration of pre-follicular cells into the female area of intersex gonad. (**M**) Infiltration of T-cells (dark brown area) into the female area of the intersex gonad. (**N**) Vasodilation in the male area of the intersex gonad. (**O**) Seminiferous tubule necrosis in the male area of the intersex gonad. (**P**) Clusters of T-cells (dark brown) resembling lymph nodules in the germinal area of the testis. (**Q**) Interstitial white fat tissue located in the ovarian component of the intersex gonad. (**R**) White fat tissue located laterally to male area of intersex gonad. (**S**) Vitellogenin (brown) in the plasma of intersex gonad blood vessels. (**T**) Vitellogenin deposition (brown) in a Sertoli cell adjacent to an ovarian follicle. **Indicators:** tunica albuginea (white star), spermatogonia (green arrowheads), Sertoli cells (red arrowheads), primary spermatocytes (Sc I), secondary spermatocytes (Sc II), spermatid–spermatozoa conversion (St-Sz), degenerating spermatogonia (blue arrowheads), oocytes (yellow arrowheads), blood vessels (black arrows), foci of necrosis (white arrow), adipocytes (black stars). **Staining**: Azan trichrome ((**A**,**B**) and magnifications inside), H-E ((**C**–**F**,**I**,**J**,**L**) and magnification inside, (**N**,**O**,**Q**)), TUNEL ((**G**,**H**) and magnification inside, (**K**)), IHC—anti-CD 3 antibody (**M**,**P**), PAS with Weigert’s iron hematoxylin (**R**), IHC—anti-vitellogenin antibody (**S**,**T**). **Scale bars**: (**A**–**C**,**H**,**L**,**N**) = 100 µm; (**D**–**F**,**G**,**J**,**K**,**O**,**S**,**T**) = 50 µm; (**I**,**M**,**P**–**R**) = 500 µm; magnifications inside (**A**,**B**,**H**,**L**) = 10 µm.

Testes were identified only in one individual among all the sampled specimens on the 1600th dph (Table 2). In contrast to the ovaries, the testicular surface was mostly smooth with rare, singular furrows and was covered with simple-squamous to low-columnar epithelium. Under the covering epithelium, fibrous (connective) tissue formed a thin tunica albuginea, which separated seminiferous tubules from the gonadal edge (Figure 3A). In some of the seminiferous tubules of the identified male, early stages of spermatogenesis were observed (magnification inside Figure 3A), and in distal (dorsal) tubules, an accumulation of spermatozoa appeared (Figure 3B). The rest of the specimens containing male germinal tissue were intersexes. In the intersex gonads, seminiferous tubules occupied the dorsal part of the gonads, while the female component developed mostly on the ventral surface (Figure 3C). However, chromatin-nucleolus oocytes in early meiotic stages were also noted inside the seminiferous tubules (Figure 3D). Spermatogenesis was observed in four out of the five intersexes (Table 2). The most developed testicular component contained seminiferous tubules with cysts of spermatocytes and a small portion of spermatids in spermiogenesis (Figure 3E). In the intersex individual with the least advanced testicular development, the lowest number of spermatogonia was observed (Table 2). In the pre-meiotic seminiferous tubules characterized by low spermatogonia density, pale Sertoli cells with deformed nuclei were observed, along with occasionally occurring cells with dark pyknotic nuclei (Figure 3F). These cells were spermatogonia, undergoing degeneration by apoptosis (Figure 3G). Significantly increased spermatogonia degeneration was observed in one intersex, while in the rest of the specimens, only singular apoptotic cells were observed (Table 2). In contrast, the most pronounced TUNEL-positive reaction was detected in the gonad with the most advanced spermatogenesis (Figure 3H).

The female component in four out of the five intersex gonads was composed of growing ovarian follicles containing perinucleolar oocytes (Figure 3C), similarly to what had been observed in the majority of the females. In one of the intersexes, an ongoing feminization process was observed, which was characterized by the presence of multiple oocytes at the chromatin-nucleolus stage, located mostly in the clusters of the folded ventral area of the gonad (Figure 3I,J). In this gonad, increased apoptosis both in the feminizing area (Figure 3K) and in pre-meiotic seminiferous tubules was observed (Table 2). In that gonad, severe infiltration of pre-follicular cells (Figure 3L) and T-cells (Figure 3M) was also observed along with vasodilation (blood vessel widening) (Figure 3N) and mild focal necrosis of the seminiferous tubules (Figure 3O). T-cells were also observed in other intersex gonads, however, to a lesser extent (Table 1). In the testes of the only identified male, T-cells occurred mainly in nodule-resembling foci (Figure 3P).

Visceral white fat tissue was present in three intersex gonads, while in the male gonads, it was absent. In one of the intersexes, adipocytes were located in the female component in the area occupied by growing ovarian follicles (Figure 3Q), similarly to what was observed in some of the females, while in the remaining two intersexes, white fat tissue was located laterally to seminiferous tubules (Figure 3R). The presence of vitellogenin was detected in only one intersex gonad, in the plasma inside blood vessels (Figure 3S) (Table 1). Additionally, a positive vitellogenin reaction was observed in a few Sertoli cells adjacent to the growing ovarian follicles (Figure 3T). In the testes of the identified male, vitellogenin was not detected.

## 4. Discussion

Although impaired gonadal development is an important issue for further sustainable sturgeon aquaculture development, surprisingly, only few pieces of research focusing on gonadal abnormalities in the sturgeon have been published to date, reflecting the insufficiency of the knowledge in this field. In the analyzed, over-4-year-old (1600 dph), RAS-reared Russian sturgeon, disturbances in gonad development were frequently observed, and some of them could have contributed to a decrease in fish fertility or even cause sterility.

The first evidence of gonadal disorders in the analyzed Russian sturgeon was a notable disproportion in the sex ratio, which was also reported for this fish stock in previous research, covering the period from 100th to 800th dph [26]. Sturgeons are gonochoristic fish species, with a sex ratio close to 1:1 [23], and intersex gonad development is considered as a pathology [30]. The female-sex biased ratio, along with the appearance of intersexes, suggests the occurrence of feminizing endocrine disruption during fish rearing. These findings are additionally supported by the detection of unspecific vitellogenin gene expression as early as on the 100th dph, in the gonads and livers of juvenile sturgeon from the analyzed fish stock [31]. Vitellogenin, beside its main function as the egg-yolk precursor protein, is also a well-known biomarker for endocrine disruption in the water environment, and its presence in males or juveniles is attributed to xenoestrogenic contamination [32,33]. In this study, vitellogenin was detected by the IHC method in three out of 15 analyzed specimens, two females and one intersex. However, in our previous study, elevated expression of vitellogenin was observed in the gonads and livers of all analyzed individuals at the 1600th dph, when quantified using qRT-PCR. However, the highest values were noted in the livers of females and intermediate levels were found in intersexes [31]. The observed discrepancy in the obtained outcomes most probably results from the lower sensitivity of the IHC method applied in this study. Even though none of the analyzed females or intersexes reached the vitellogenic stage of development, it is suggested that low plasma vitellogenin concentrations can be detected in pre-vitellogenic sturgeon gonads, which are correlated with the initiation of yolk precursor endocytosis by the oocytes [23]. Therefore, the occurrence of vitellogenin protein in blood plasma and some perinucleolar oocytes, both observed in this study, can be attributed to the onset of the early vitellogenic phase. On the other hand, the presence of vitellogenin protein in some Sertoli cells in this study may be the cause of the previously detected unspecific vitellogenin expression in the sturgeon testes [31,34]. However, the source of the low unspecific vitellogenin expression in the ovaries of the 1600th dph females, reported by Fajkowska et al. [31], is still unknown. This unspecific expression can be caused by estrogenic stimulated adipocytes, as it was shown that white adipose tissue in 17β-estradiol-treated female zebrafish (*Danio rerio*) expressed low levels of vitellogenin [35]; however, the insufficient sensitivity of the applied IHC method may not have enabled its detection in this study. Nevertheless, further research is needed to confirm these findings and also to investigate the cause of unspecific vitellogenin expression in sturgeon gonads.

The most detrimental histopathology observed in the present research, except intersex gonadal development, concerned ovarian follicle shortage in the ovaries with profound intraovarian white fat tissue development. The etiology of ovarian fat deposition in sturgeons is unclear [36], although its development is highly undesirable, as fatty ovaries tend to produce a smaller yield of inferior-quality roe, necessitating additional labor in caviar processing [36,37]. In the analyzed ovaries with the most severe fat overgrowth, ovarian follicles occupied only a small percentage of the gonadal area, carrying about 85% fewer follicles than the gonads with no fat. Moreover, it needs to be noted that the given values of follicle density in fatty ovaries are underestimated, as they were counted only in the folded gonadal surface, in which a few residual follicles were still observed, while the rest of gonadal volume was occupied by white fat tissue. This means that the most-affected females were almost sterile. Ovarian sterility resulting from the substitution of the germinal tissue with the adipose tissue was noted not only in the farmed sturgeon but also in wild specimens from the Ob river [38,39]. The significant decrease in follicles in the analyzed females could have resulted either from an insufficient number of germinal cells in early developmental stages or their increased degeneration during the following periods of gonadal development. The second hypothesis is more likely, as the former study concerning gonadal development conducted on this fish stock from one day after hatching showed no signs of inhibition of the germinal cell proliferation at early stages of gonadogenesis [28]. On the other hand, the second hypothesis might be unfounded, due to a low number of early atretic follicles observed in the ovaries with fat overgrowth at the 1600th dph. Nevertheless, the progressive loss of oocytes in these females might have been initiated in the earlier stages of gonadal development, the way it was observed in the female with ovarian nests in this study. To date, it is not known if the development of intraovarian fat tissue plays a vital role in germinal tissue degeneration or if it simply emerges after this process and expands in the area previously occupied by reproductive tissue. However, Fedorovykh et al. [40] suggested that in mature sturgeons, high ovarian adiposity may be connected to reproductive problems, such as reduced ovulation and fertility. In sturgeons, follicular atresia is a process that is strongly related by testosterone and 17β-estradiol plasma concentrations [41]. White fat tissue is not only a passive reservoir for energy storage, but also, apart from the gonads and adrenal glands, it is an important endocrine organ for both metabolism and the secretion of sex steroids. Due to cytochrome P450-dependent aromatase and 17βHSD activity, adipose tissue mediates the conversion of androgens to estrogens and of weak sex hormones to their more potent counterparts [42]. Thus, adipose tissue overgrowth may play an important role in testosterone and 17β-estradiol regulation, affecting oocyte development and inducing follicular atresia. Under RAS conditions, an energetic surplus can be easily achieved, which can result in increased visceral fat deposition and subsequently lead to an imbalance of sex hormones and disturbed gonadal development in sturgeons. A reduction in the ovarian adipocyte size can be achieved by lowering dietary fat at the pre-pubertal stage of sturgeon development [43]. Nevertheless, the mutual interaction between ovarian adipogenesis and oocyte loss should be urgently researched in the future, as increased degeneration of germinal cells in the early stages of gonadal development will have a detrimental effect on fish fecundity.

Follicular atresia is an important aspect of the fish ovarian cycle and physiology, playing an essential role in the regulation of the oocyte number that attain ovulation [44]. In fish, follicular atresia occurs under normal conditions due to seasonal changes—most frequently, however, it is observed in the post-spawning period [45,46]. Contrarily, increased atresia, particularly of previtellogenic follicles, can indicate a pathological condition and has been associated with exposure to environmental contaminants [47]. Follicles may become atretic at any stage of oogenesis [46]. However, not much research focuses on oocyte degeneration in the previtellogenic stages of gonadal development, even though the significance of this phenomenon is indisputable, especially in sturgeon aquaculture. In the previtellogenic ovaries of the analyzed sturgeon, numerous follicles in different stages of atresia were recognized. Oogonia and small oocytes were shown to have undergone atresia by apoptosis, vanishing completely through progressive degeneration. Therefore, increased atresia in ovaries at early stages of oogenesis may not be detected in later phases of development. In contrast to the early meiotic oocytes, the elimination of larger perinucleolar oocytes was caused by autophagy. Oocytes that underwent this process showed differential morphology; thus, multiple molecular mechanisms are suspected of participating in this process. Moreover, the observed oocyte autophagy was accompanied by increased apoptosis of the follicular cells surrounding the oocytes, which suggests their important role in follicular atresia [48]. This also implies that both apoptosis and autophagy interact during follicular atresia in sturgeons, as has been shown for some other fish species [49]. Follicular atresia is frequently associated with environmental stress or changes in hormonal levels [50]. Considering that female germinal cell degeneration in the studied fish coexisted with other gonadal impairments, such as intersex occurrence, it is likely that all of them originated from hormonal imbalance, as increased oocyte degradation and resorption at any point of development is also a well-known ovarian response to endocrine disruption [51]. The potential sources of endocrine disrupting chemicals (EDCs) in indoor RAS supplied by tap water are limited. However, Wee and Aris [52] indicate that tap water might be a source of EDCs. This might be related to groundwater contamination or ineffective water treatment in wastewater treatment plants. Background contamination differs by the location, while the highest concentrations are reported for developed countries. Therefore, in particular cases, water can be a source of endocrine-active compounds. The next important source of EDCs in sturgeon farming is fish diets. The estrogenic effect of sturgeon feed, measured by the induction of vitellogenin synthesis in the liver or the increase in vitellogenin in blood plasma, was previously reported by Pelissero et al. [53,54] and Fajkowska et al. [55]. Phytoestrogens are considered the main source of EDCs in sturgeon feeds [53]. These compounds occur in high concentrations in legumes, especially in the soybean [56], and therefore, they are commonly found in high concentrations in fish feeds [57], including sturgeon commercial diets [29]. Phytoestrogens, due to their ability to bind and interact with estrogen receptors, mimic endogenic estrogens [58], and therefore, they may interfere with fish sex differentiation [59,60,61]. For sturgeons, with their long gonadal development, chronic exposure to EDCs may have particularly negative consequences on reproduction. The presence of soybean phytoestrogens—namely, genistein, daidzein and the highly estrogenic phytoestrogen metabolite equol—was previously detected in the livers and blood of sturgeon originating from the analyzed sturgeon stock. The same phytoestrogens were also detected in substantial amounts in fish feeds, indicating that diet is an important source of sturgeon exposure to EDCs [29]. However, to date, there has been a striking lack of knowledge on the effects of dietary phytoestrogens on sturgeon gonad differentiation. Phytoestrogens are known to induce histological alterations in the gonadal tissue of treated fish. Two highly potent phytoestrogens, genistein and equol, were reported to cause delayed oocyte maturation and the induction of oocyte atresia, combined with the proliferation of somatic stromal tissue in medaka (*Oryzias latipes*) [61]. Genistein was also shown to be very potent in altering endogenous hormone levels in fish, causing increased ovarian estradiol production and decreased levels of circulating testosterone [62]. Moreover, phytoestrogens were also shown to be conducive to adipogenesis in fish. Data obtained by Cleveland and Manor [63] suggested that genistein, as well as estradiol, increased the liver expression of fatty-acid- and lipid-binding protein synthesis genes in the rainbow trout (*Oncorhynchus mykiss*). On the other hand, phytoestrogens may not be the only endocrine disruptors in fish diets, since multiple potential EDCs in feeds may not have been identified as of yet. For example, fish oil, one of the main components of all fish diets, was shown to have both estrogenic and anti-androgenic activity due to the presence of polychlorinated biphenyls (PCB) and various DDT metabolites, which can influence sex hormone receptors [64]. Moreover, water contamination with PCB and common breakdown products of DDT-DDE, was previously suggested to cause the occurrence of 29% of intersexes in the Mississippi River shovelnose sturgeon (*Scaphirhynchus platyorynchus*) [65].

Contrary to that in ovaries, the analysis of the gonadal impairment in testicular tissue in this study was difficult as only one male was recognized among 15 sampled Russian sturgeon, while the rest of the male specimens probably feminized, producing intersexes, which is already a pathological condition. However, it is worth noticing that in none of the analyzed specimens, at an age above 4 years old, did the testicular component reach sexual maturity. Normal Russian sturgeon males mature at the age of 4–5 years old [66,67,68]. The male component of the intersex gonad may not have developed at the same rate as normal testes, although Jackson et al. [66] reported that five-year-old Russian sturgeon intersexes were characterized by mature testicular tissue, whereas oocytes were in the previtellogenic stage of development. Therefore, the inhibition of spermatogenesis can be suspected in the analyzed fish, although the general lack of males in the analyzed stock does not allow the confirmation of this thesis. In contrast to in the ovaries, in the testes or testicular tissue of the intersex specimens, only singular spermatogonia in pre-meiotic seminiferous tubules underwent elimination, showing morphology typical of apoptotic cells. An increased number of apoptotic spermatogenic cells was observed particularly in meiotic seminiferous tubules, yet this phenomenon typically occurs with the first wave of spermatogenesis in vertebrate males during sexual development [69]. Only one intersex showed increased apoptosis in premeiotic-seminiferous tubules and in the area where male and female germinal tissue components are directly adjacent. In the adjoined region, some necrotic seminiferous tubules were also sporadically found, which can be related to the local rearrangement of germinal tissue during feminization. In such areas, an infiltration of both pre-follicular and T-cells was also observed. The local excess of both cell types may indicate an endocrine-related histopathology, as multiple EDCs can induce pre-follicular cell hyperplasia/hypertrophy [51]. The balance of the proliferation/apoptosis of lymphocytes in fish can also be affected by estrogens and estrogen-like disruptors [70]. However, in the case of fish, there is no clear data consistency on lymphocyte proliferation in response to estradiol and other sex-steroid EDCs [70]. Nevertheless, some studies conducted on the mouse model suggest that the administration of a low dose of estradiol enhances primary antigen-specific T-cell proliferative responses [71]. The accumulation of lymphocytes in the ovaries was also observed in both wild and hatched sterlet (*Acipenser ruthenus*) and Siberian sturgeons. This phenomenon was shown to be an indirect indicator of pathological processes in sturgeon ovaries and was assigned to increased oocyte resorption [38,39]. Moreover, obesity and overweightness may cause chronic low-grade inflammation [72], which could have caused leukocyte infiltration in the gonads of the analyzed fish. However, a straightforward correlation between the amount of ovarian fat and the increased number of leukocytes in the analyzed gonads was not found.

Another potential cause of the observed gonadal disruption in the analyzed sturgeon might have derived from genetic disorders. Fish sterility and semi-sterility often occur as a result of interspecific hybridization [73]. Moreover, in the hybrid progeny of sturgeons with different parental polyploidy, apoptosis of early meiotic oocytes was detected [74]. On the other hand, Jackson et al. [66] demonstrated, based on cytochrome b and D-loop genetic analyses, that Russian sturgeon intersex appearance was not caused by interspecific hybridization. Interestingly, in that study, the plasma steroid profile (11-ketotestosterone and 17β-estradiol) and βFSH gene expression level in intersex fish were similar to those of a male, although ova-testes with a largely prevailing female component were observed [66]. The analyzed Russian sturgeon were pure line; thus, a genetic disorder seems not to have been the initiating factor of the observed impairments. However, the molecular pathways of gonadal development in the analyzed fish were very likely disturbed in response to the environmental condition.

The abovementioned potential causes of the observed gonadal pathologies in the analyzed sturgeon do not exhaust the topic, as there is a wide spectrum of genetic and environmental factors that can affect gonadal development, leading to the observed gonadal impairments. The identification of the potential ones is a particularly difficult task, especially due to the fact that sex determination mechanisms in sturgeons are still insufficiently recognized [75]. Sturgeon RAS farming has many advantages; however, particular emphasis should be placed on proper sturgeon gonadal development under RAS conditions, as disturbed gonadal development or differentiation can lead to a decline in fish reproduction capacity. In this regard, further research should be focused on identifying the causes of gonadal disruption in sturgeons and their effect on the reproduction of these prized fish.

## 5. Conclusions

In over-4-year-old (1600 days post-hatching) Russian sturgeon reared in an indoor RAS, multiple gonadal impairments were observed, some of which can significantly affect reproduction capacity. The most detrimental of the observed histopathologies might originate from endocrine disruption, although its sources need to be further investigated.

## Figures and Tables

**Table 1:** Characteristic of the ovarian component of recirculating aquatic system (RAS)-reared Russian sturgeon on the 1600th day after hatching.

No.	Sex	Type of Gonad	Stage of Development ^†^	No. of Ovarian Follicles (mm^2^)	Early Atretic Follicles (%)	Intraovarian Fat	Lymphocyte Infiltration	Presence of Vitellogenin	Figures
1	female	ovary	2	6.97 ± 1.67 ^c^	6.52 ± 7.72 ^c,d^	***	-	-	Figure 1D and Figure 2A,I,L
2	female	ovary	2	44.11 ± 3.16 ^a^	12.12 ± 5.85 ^a^	-	-	-	Figure 1A and Figure 2B,K,N,O
3	female	ovary	2	19.40 ± 3.79 ^d^	6.50 ± 4.02 ^c,d^	**	*	blood plasma and small oocytes in PNS	Figure 2C,D
4	female	ovary	2	20.10 ± 3.69 ^d^	6.76 ± 5.65 ^c,d^	*	*	-	Figure 2H,P,S
5	female	ovary	2	23.72 ± 3.24 ^b^	8.35 ± 2.23 ^a,c^	**	-	-	Figure 2M
6	female	ovary	1	NC	NC	***	***	-	Figure 2E,Q,R,T
7	female	ovary	2	11.25 ± 4.45 ^e^	1.81 ± 2.9 ^b^	***	*	-	Figure 2G
8	female	ovary	1/2	NC	NC	**	*	blood plasma and small oocytes in PNS	Figure 2F
9	female	ovary	2	14.08 ± 4.39 ^e^	2.83 ± 2.58 ^b,d^	***	-	-	Figure 2J
10	intersex	ova-testis	1/2	NC	NC	-	***	-	Figure 1B and Figure 3D,I–O
11	intersex	testis-ova	2	NC	NC	*	**	blood plasma and Sertoli cells	Figure 1C and Figure 3C,Q,S,T
12	intersex	testis-ova	2	NC	NC	-	**	-	Figure 3F,G,R
13	intersex	ova-testis	2	NC	NC	-	*	-	Figure 3E,H
14	intersex	testis-ova	2	NC	NC	-	*	-	-
15	male	testis	-	-	-	-	**	-	Figure 3A,B,P

Mild (*), moderate (**), severe (***); Not counted (NC); Perinucleolar stage (PNS). The superscript lower-case letters indicate statistically significant (*p* < 0.05) differences. ^†^ classification according to Webb et al. [17]: 1—differentiation; 2—pre-vitellogenic.

**Table 2:** Characteristics of the testicular component of RAS-reared Russian sturgeon on the 1600th day after hatching.

No.	Sex	Type of Gonad	Stage of Development ^†^	Spermatogenic Tubules (%)	No. of Spermatogonia (mm^2^)	Degenerating Spermatogonia (%)
10	intersex	ova-testis	2/3	0.36 ± 0.81 ^c^	1668 ± 1431 ^b^	32.5 ± 41.7 ^a^
11	intersex	testis-ova	2/3	30.79 ± 10.85 ^b^	2051 ± 979 ^b^	9.17 ± 24.47 ^b^
12	intersex	testis-ova	2	0.00 ± 0.00 ^c^	1020 ± 735 ^a^	14.75 ± 26.22 ^b^
13	intersex	ova-testis	4	100 ± 0.00 ^a^	NC	NC
14	intersex	testis-ova	2/3	0.85 ± 1.44 ^c^	1458 ± 562 ^a,b^	1.00 ± 4.47 ^b^
15	male	testis	2/3	3.26 ± 1.99 ^c^	1845 ± 918 ^b^	7.50 ± 14.53 ^b^

Not counted (NC). The superscript lower-case letters indicate statistically significant (*p* < 0.05) differences. ^†^ classification according to Webb et al. [17]: 2—pre-meiotic; 3—onset of meiosis; 4—meiotic.
